# Bone fragility and osteoporosis in children and young adults

**DOI:** 10.1007/s40618-023-02179-0

**Published:** 2023-09-05

**Authors:** M. M. Formosa, M. A. Christou, O. Mäkitie

**Affiliations:** 1 Department of Applied Biomedical Science, Faculty of Health Sciences, University of Malta, Msida, Malta; 2 Center for Molecular Medicine and Biobanking, University of Malta, Msida, Malta; 3 Department of Internal Medicine, Erasmus University Medical Center, Rotterdam, The Netherlands; 4 Department of Endocrinology, School of Medicine, University of Ioannina, Ioannina, Greece; 5 Department of Hygiene and Epidemiology, School of Medicine, University of Ioannina, Ioannina, Greece; 6 Children’s Hospital, University of Helsinki and Helsinki University Hospital, Helsinki, Finland; 7 Research Program for Clinical and Molecular Metabolism, Faculty of Medicine, University of Helsinki, Helsinki, Finland; 8 Folkhälsan Research Centre, Folkhälsan Institute of Genetics, Helsinki, Finland; 9 Department of Molecular Medicine and Surgery, Karolinska Institutet, and Clinical Genetics, Karolinska University Hospital, Stockholm, Sweden

**Keywords:** Early-onset osteoporosis, Bone mass, DXA, Osteogenesis imperfecta, Secondary osteoporosis, Fragility fractures, Genetic testing, Idiopathic osteoporosis

## Abstract

Osteoporosis is a metabolic bone disorder which increases fragility fracture risk. Elderly individuals, especially postmenopausal women, are particularly susceptible to osteoporosis. Although rare, osteoporosis in children and young adults is becoming increasingly evident, highlighting the need for timely diagnosis, management and follow-up. Early-onset osteoporosis is defined as the presence of a low BMD (Z-score of ≤ −2.0 in individuals aged < 20 years; T-score of ≤ −2.5 in those aged between 20 to 50 years) accompanied by a clinically significant fracture history, or the presence of low-energy vertebral compression fractures even in the absence of osteoporosis. Affected children and young adults should undergo a thorough diagnostic workup, including collection of clinical history, radiography, biochemical investigation and possibly bone biopsy. Once secondary factors and comorbidities are excluded, genetic testing should be considered to determine the possibility of an underlying monogenic cause. Defects in genes related to type I collagen biosynthesis are the commonest contributors of primary osteoporosis, followed by loss-of-function variants in genes encoding key regulatory proteins of canonical WNT signalling (specifically *LRP5* and *WNT1*), the actin-binding plastin-3 protein (encoded by *PLS3*) resulting in X-linked osteoporosis, and the more recent sphingomyelin synthase 2 (encoded by *SGMS2*) which is critical for signal transduction affecting sphingomyelin metabolism. Despite these discoveries, genetic causes and underlying mechanisms in early-onset osteoporosis remain largely unknown, and if no causal gene is identified, early-onset osteoporosis is deemed idiopathic. This calls for further research to unravel the molecular mechanisms driving early-onset osteoporosis that consequently will aid in patient management and individualised targeted therapy.

## Introduction

Osteoporosis is a progressive, multifactorial systemic skeletal disease characterised by low bone mass, microarchitectural deterioration of bone tissue and reduced bone strength that culminates in increased fracture risk [1, 2]. Fractures of the hip and vertebrae are the most common, debilitating and costly and occasionally can lead to death in 20% of affected individuals within the first year of fracture [3–6]. Although osteoporosis is considered a disease of the elderly, affecting particularly postmenopausal women, increased clinical attention is being given to low bone mass disorders in children and young adults —whether primary or secondary in nature especially with the advent of improved or new diagnostic techniques [7, 8].

Bone is a physiologically dynamic organ exhibiting exceptional properties, ranging from mechanical to metabolic and endocrine functions. It is a complex living tissue encompassing a variety of different cells (osteoblasts, osteocytes, bone lining cells and osteoclasts) within a mineralised matrix, all of which contribute towards maintaining a healthy bone status [9]. Mechanically, the skeleton supports the body and protects the vital organs. Metabolically, this endocrine organ is primarily a major source of minerals, growth factors, hormones and fatty acids. Bone is composed of an inorganic portion (50–70%) consisting of hydroxyapatite (Ca_10_(PO_4_)_6_(OH)_2_), an organic matrix (20–40%) being chiefly made up of type I collagen, water (5–10%) and impurities [9, 10]. The degree of mineralisation influences mechanical resistance and rigidity of bones, enabling them to withstand compression forces and loading, whereas the collagenous matrix allows for elasticity and movement. At the microarchitectural level, bone consists of cortical (making up approximately 80% of bone) or trabecular bone differing in structural organisation, function and site distribution. Cortical bone is made up of densely packed collagen fibrils forming concentric bone lamellae parallel to and around central Haversian canals through which blood and lymphatic vessels, nerves and connective tissue flow. Trabecular bone is composed of irregularly organised rod and plate-like networks of trabeculae forming 3D lattices arranged along the lines of stress. Despite constituting 20% of the skeleton, trabecular bone harbours a higher surface area relative to cortical bone and undergoes more active remodelling making it more susceptible to pathogenesis [11, 12].

The precise and proper balance between bone formation and resorption is imperative in the shaping and development of bones, maintaining the integrity of the skeleton and in systemic mineral homeostasis. Bone modelling is prominent in childhood and helps to define bone structure, shaping, expansion and movement through space in response to the combined effect of mechanical loading, hormonal control and genetic factors affecting osteoblast and osteoclast function [13, 14]. Conversely, remodelling is a self-regeneration process involving the coordinated action or ‘coupling’, between osteoblastic bone formation and osteoclast-mediated bone resorption, which must be timely and quantitatively balanced by paracrine and endocrine factors and immune cells. Remodelling takes place in stages starting by osteoclast activation and resorption of existing damaged bone, reversal whereby osteoblasts are recruited to the bone surface and bone formation by osteoblasts that lay down osteoid which becomes mineralised forming mature bone [11, 13]. The coupling between resorption and formation is balanced and relatively stable during peak adult mass. However, it decreases over time with ageing increasing the risk of low bone mass and fracture susceptibility [11, 13, 15].

In this review, we describe the recent definition of early-onset osteoporosis and its aetiology, the clinical diagnostic evaluation including genetic testing methods to confirm the presence of an underlying monogenic cause and treatment options for affected individuals.

## Definition of early-onset osteoporosis

Bone mineral density (BMD) measurement by dual-energy X-ray absorptiometry (DXA) can be used to diagnose osteoporosis in postmenopausal women and men aged > 50 years. The World Health Organization defines osteoporosis in these populations as a BMD at the spine, hip or forearm of 2.5 or more standard deviations below the young adult mean (T-score ≤ -2.5) [16–18]. Additionally, in all cases of unusual fracture, pathologies such as osteomalacia (e.g. due to severe vitamin D deficiency, hypophosphataemia), malignancy or fibrous dysplasia should be ruled out [7, 17–21]. Subsequently, any fracture of low-to-moderate energy trauma (aside from a fracture of the digits, skull or face) that occurs from a standing height or less can be considered a low-trauma or fragility fracture [17, 22]. Such individuals may have decreased bone strength and may be considered to have osteoporosis, irrespective of BMD.

However, the diagnostic guidelines of osteoporosis in children and young adults are different (Table 1). The International Society for Clinical Densitometry (ISCD) recommends the use of BMD Z-scores in these populations (compared with age-matched norms) [23, 24]. In premenopausal women and men aged < 50 years, a Z-score ≤ − 2.0 is interpreted as below the expected range for age and a Z-score > − 2.0 as within the expected range for age [25, 26]. In this age group, osteoporosis diagnosis should not be based only on low BMD, but also on a history of low-trauma fracture or a secondary cause of osteoporosis. In children, the values should be properly adjusted for short stature and/or delayed or advanced timing of puberty [23]. In the absence of vertebral compression fractures, the diagnosis of osteoporosis is indicated by the presence of both a clinically significant fracture history and BMD Z-score ≤ − 2.0. A clinically significant fracture history is one or more of the following: (1) two or more long bone fractures up to age of 10 years, (2) three or more long bone fractures up to age of 19 years [23, 24]. Additionally, in this age group, one or more vertebral compression fractures is indicative of osteoporosis, in the absence of local disease or high-energy trauma, even if the BMD Z-score is not subnormal. The International Osteoporosis Foundation (IOF) defines low bone mass as a Z-score of ≤ − 2.0 in subjects aged < 20 years and in those aged > 20 years with delayed puberty [27]. The IOF suggests the use of a T-score < − 2.5 to define osteoporosis in subjects aged 20–50 years in association with a low-trauma fracture history or a secondary cause of osteoporosis.

**Table 1:** Definition of osteoporosis in children and young adults

**International society for clinical densitometry (ISCD)**
*Children* ≥ 1 vertebral compression fractures in the absence of local disease or high-energy trauma, or Clinically significant fracture history and BMD Z-score ≤ − 2.0 with ≥ 2 long bone fractures up to age 10 years and/or ≥ 3 long bone fractures up to age 19 years
*Premenopausal women and men aged* < *50 years* BMD Z-score ≤ − 2.0 and low-trauma fracture or secondary cause of osteoporosis
**International Osteoporosis Foundation (IOF)**
*Young adults aged 20–50 years* T-score ≤ − 2.5 and low-trauma fracture or secondary cause of osteoporosis

*BMD* bone mineral density

## Aetiology of early-onset osteoporosis: identifying the underlying cause

Low bone mass may be related to either inadequate peak bone mass acquisition and/or ongoing bone loss. BMD depends primarily upon achievement of peak bone mass which is defined as the maximum BMD achieved by age 40 years [28, 29]. Importantly, 95–100% of peak bone mass is acquired by the late teen years [30–33] making this a crucial period for the proper formation of a robust musculoskeletal system.

**Fig. 1:**
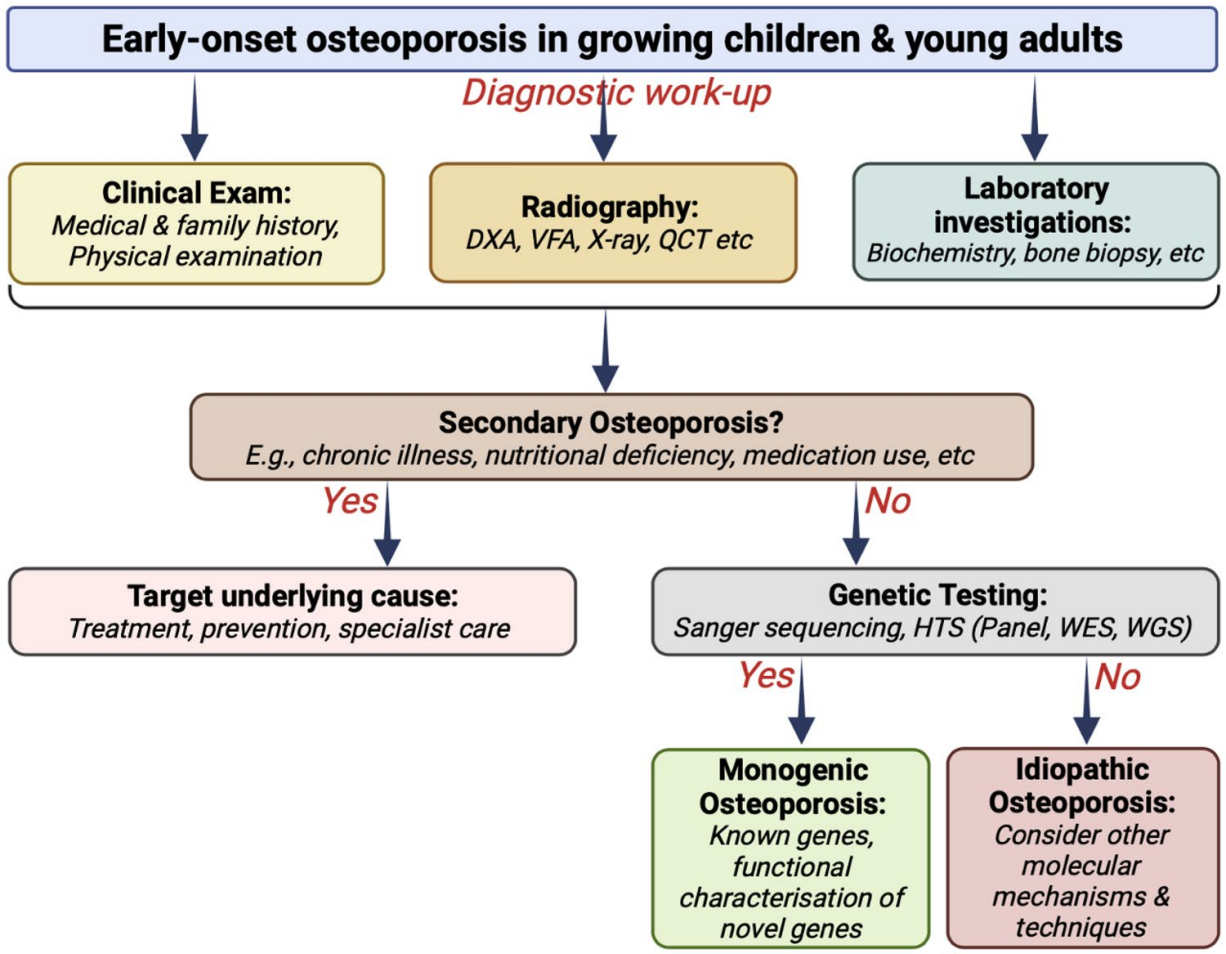
Flowchart showing the diagnostic workup of a growing child or young adult with suspected early-onset osteoporosis. *DXA* dual-energy X-ray absorptiometry, *VFA* vertebral fracture assessment, *QCT* quantitative computed tomography, *HTS* High-throughput sequencing, *WES* whole-exome sequencing, *WGS* whole-genome sequencing. Figure created using BioRender (https://biorender.com)

Bone loss and/or fragility fractures in children and young adults can be attributed to a secondary cause which needs to be carefully looked for. If no such cause is identified, bone fragility may then be regarded primary and potentially related to rare gene variants [34]. If there is still no apparent aetiology, bone loss and/or fractures are considered idiopathic (Fig. 1).

### Secondary causes

Many secondary risk factors are similar to those for postmenopausal osteoporosis and osteoporosis in men. Table 2 includes secondary causes of osteoporosis in children and young adults and some of the more common conditions are described below.

**Table 2:** Secondary causes of low bone mass/fractures in children and young adults

**Endocrine diseases**	**Medications**
Cushing’s syndrome (ACTH, non-ACTH dependent)	Anticonvulsants
Diabetes mellitus	Aromatase inhibitors
GH deficiency	Chemotherapy
Hypercalciuria	Depot medroxyprogesterone acetate
Hyperparathyroidism	Excess levothyroxine
Hyperprolactinaemia	Glucocorticoids
Hyperthyroidism	GnRH agonists
Hypogonadism (hypogonadotropic, hypergonadotropic)	Heparin
Hypophosphatasia	Immunosuppressants
Hypophosphataemia	Proton pump inhibitors
Vitamin D and/or calcium deficiency	SSRI
	Thiazolidinediones
**Haematologic diseases**	**Metabolic diseases**
Bone marrow transplantation	Gaucher’s disease
Haemophilia	Glycogen storage disease
Hereditary haemochromatosis	Homocystinuria
Leukaemia	Mucopolysaccharidoses
Lymphoma	**Malnutrition/malabsorption**
Mastocytosis	Anorexia nervosa
Multiple myeloma	Celiac disease
Thalassemia major	Gastrointestinal surgery
**Chronic inflammatory diseases**	**Other**
Inflammatory bowel disease	Alcoholism
Liver diseases	Cystic fibrosis
Lung diseases	Duchene muscular dystrophy
Kidney diseases	Excessive exercise
Rheumatic diseases	HIV
Skin diseases	Pregnancy and lactation

*ACTH* adrenocorticotropic hormone, *GH* growth hormone, *GnRH* gonadotropin-releasing hormone, *HIV* human immunodeficiency virus, *SSRI* selective serotonin reuptake inhibitor

### Chronic inflammatory diseases

The aetiology of low BMD and fragility fractures in chronic inflammatory diseases, such as rheumatic diseases, lung diseases, inflammatory bowel disease, liver and kidney diseases, and skin diseases includes effects of the disease itself, systemic inflammation, glucocorticoids use, low body weight, malabsorption, low physical activity, delayed puberty and/or secondary amenorrhoea [17, 34, 35].

### Glucocorticoid use

The negative effects of the glucocorticoids on bone include increased apoptosis of osteoblasts and osteocytes, decreased apoptosis of osteoclasts, negative effects on muscle function, decreased calcium absorption in the gut and decreased calcium re-absorption in the kidney [17]. The American College of Rheumatology advises to assess clinical fracture risk in all children and young adults within 6 months of starting glucocorticoids and to perform DXA in adults aged < 40 years when there is a history of osteoporotic fracture or other risk factors for fracture [36, 37]. In adults aged > 40 years, FRAX should be used with glucocorticoid dose correction and BMD should be tested within 6 months of starting glucocorticoids.

### Oestrogen deficiency

Examples of premenopausal oestrogen deficiency include Hypogonadotropic hypogonadism due to low body weight, anorexia nervosa, excessive exercise, hyperprolactinaemia and hypopituitarism and Hypergonadotropic hypogonadism (premature ovarian insufficiency) due to chromosomal abnormalities (e.g. Turner syndrome, fragile X syndrome), chemotherapy, radiation and autoimmune diseases [38, 39].

### Pregnancy and lactation

Normal pregnancy can be associated with bone loss of approximately 3–5% at the spine and hip [40–42], significant decline only at the trochanter [41], or stable BMD [43]. Lactation has more consistent effects and is associated with bone loss of 3–10% at the spine and hip seen over 3–6 months [44, 45]. Bone loss is related to duration of lactation and amenorrhoea and is not prevented by calcium supplementation [46]. Parathyroid hormone-related protein (PTHrP), which is secreted by the mammary gland and controls calcium mobilisation from bone [47, 48], as well as oestrogen deficiency, may be involved in bone loss. Although there is a loss of bone mass in pregnancy and lactation, physiologically there is a partial recovery. Recovery from lactation-associated bone loss may continue for 18 months or longer [49, 50]. It has been found that parity and lactation have no adverse associations with clinical fragility or radiographic vertebral fractures, or the rate of BMD decline over 10 years [51].

Pregnancy and lactation-associated osteoporosis (PLO) is a rare condition in which women present with fractures, often vertebral, in the third trimester of pregnancy or in the early postpartum period [52, 53]. In most women, no known cause of osteoporosis is found [54]. Evaluation for secondary causes of osteoporosis should be undertaken. Skeletal fragility in PLO may result from abnormal pregnancy-related bone changes. In some women, an underlying genetic predisposition may be identified, suggesting a pre-existing monogenetic form of osteoporosis with an exacerbation due to pregnancy [55]. Abnormal osteoblast function or other bone formation defects may contribute to the pathophysiology of PLO [56]. Some patients will improve spontaneously, while others will need treatment with antiresorptive or anabolic treatment [39]. There is an increased risk of fracture recurrence (overall and within the context of another pregnancy); 24% of patients with PLO followed for 6 years had subsequent fractures, most were vertebral fractures and number of fractures at diagnosis predicted subsequent risk [57].

## Genetic causes of osteoporosis

### Osteogenesis imperfecta and other monogenic bone fragility disorders

Genetic factors play an important role in osteoporosis and determine up to 80% of BMD [2, 58]. Several contributing genes have been identified in genome-wide association studies (GWAS) and the risk is thought to depend on several gene variants, each with modest effect sizes [59–62]. In monogenic forms, osteoporosis is caused by a single variant in a gene that has a major role in the skeleton [63]. The most recent nosology of genetic skeletal disorders lists altogether 55 genetic and clinical entities with skeletal fragility [64]. Osteogenesis imperfecta (OI) is the most common of these monogenic disorders with skeletal fragility; it is usually caused by mutations in the genes regulating extracellular matrix, especially type I collagen [65–70]. Apart from extracellular matrix defects, other mechanisms may also lead to skeletal fragility. These include impaired osteoblast and osteoclast function, defective matrix mineralisation and defects in calcium and phosphate homeostasis.

Only a small number of genetic entities presenting with early-onset osteoporosis without the classical features of OI or syndromic features have been recognised [64]. These genetic forms are summarised in Table 3. The WNT signalling pathway plays a major role in skeletal homeostasis [71]. Biallelic mutations in the WNT receptor, *LRP5*, lead to severe childhood-onset osteoporosis and blindness, while heterozygous loss-of-function variants lead to milder forms of osteoporosis, often presenting later in childhood or in adulthood [72]. It has become apparent that WNT1 is the key ligand for the canonical WNT signalling pathway in bone [73, 74]. Similar to *LRP5*, biallelic and monoallelic *WNT1* variants lead to different degrees of skeletal fragility. Children with biallelic *WNT1* variants present with severe skeletal fragility mimicking OI type III, while heterozygous *WNT1* variants lead to an osteoporosis phenotype that manifests often only later in childhood or in adulthood [75–79].

**Table 3:** Genes linked to early-onset osteoporosis

Gene	OMIM	Inheritance	Mutation	Protein	Function
*LRP5*	259770 166710	AR, AD	LoF	Low-density lipoprotein-related receptor 5	WNT signalling
*WNT1*	615220	AR, AD	LoF	Wingless-type MMTV integration site family, member 1	WNT signalling
*PLS3*	300910	XL	LoF	Plastin 3	Formation of F-actin bundles
*SGMS2*	126650	AD	LoF	Sphingomyelin synthase 2	Mineralisation
*ARHGAP25*	610587	AD	LoF	Rho GTPase-activating protein 25	Bone cell function and bone metabolism

*AR* autosomal recessive, *AD* autosomal dominant, *XL* X-linked, *LoF* loss of function

In 2013, mutations in *PLS3* were identified as a cause for osteoporosis [80]. Due to the gene’s X-chromosomal location, *PLS3* mutations affect males more and earlier than females, but mutation-positive females may also develop symptomatic osteoporosis already in childhood or later in adulthood [81, 82]. Regarding the nature of reported variants, the studies have identified both missense and nonsense variants but also partial or total deletions of the gene, as well as a partial duplication of the gene in individuals with early-onset osteoporosis [83]. The gene codes for Plastin3, an actin-binding and actin-bundling protein involved in cytoskeleton remodelling [80]. The function of PLS3 in bone is still unknown. Plastin3 may be involved in the process of mechanosensing by osteocytes [84]. Recent findings indicate that PLS3 may also play a role in bone mineralisation [85].

Several other novel forms of monogenic osteoporosis have been recently described, for example those caused by variants in *SGMS2* and *ARHGAP25* [86, 87]. Individuals with a heterozygous mutation in *SGMS2*, encoding sphingomyelin synthase 2 (SMS2), had since childhood multiple fractures and often calvarial hyperostotic lesions [87]. Bone biopsies showed low bone volume, impaired matrix mineralisation and abnormal bone lamellarity with areas of 'woven bone' and a significantly disturbed osteocyte canalicular network [88]. Several subjects displayed in addition to osteoporosis, neurological symptoms, e.g. transient facial nerve palsy, suggesting that these extra-skeletal manifestations may be a distinctive feature of SGMS2-related osteoporosis [87]. The recurrent *SGMS2* p.Arg50* stop-gain variant was present in four unrelated families and has since then been reported in several additional cases [89, 90]. In two families, a missense mutation in the same gene led to a much more severe disorder with skeletal dysplasia, significant calvarial hyperostosis, severe short stature and skeletal fragility since early infancy [87].

Despite these discoveries, genetic causes and underlying mechanisms in early-onset osteoporosis remain largely unknown. The spectrum of genetic and cellular pathology is complex [34] and hence patient management also requires individualised treatment strategies. To optimise management, the characteristic skeletal and extra-skeletal pathology and the disease course in each genetic form need to be elucidated.

### Idiopathic osteoporosis

Children and young adults experiencing repetitive fragility fractures in the presence of a low BMD are primarily investigated for an underlying secondary cause or a monogenic defect in known or novel genes. Only when such causes are appropriately ruled out should idiopathic osteoporosis be considered. Indeed, some of the previously thought idiopathic cases turned out to be monogenic in nature when more extensive genetic testing became available, particularly during the high-throughput sequencing (HTS) era [91, 92].

Idiopathic osteoporosis is likely to be a heterogeneous disorder given the fact that bone remodelling and bone formation rate can be high, normal, or low [93]. In fact, several studies exploring the potential genetic causes for idiopathic osteoporosis have shown a variable monogenic aetiology [94, 95]. It is likely that parallel to increasing genetic knowledge, improved genetic tools and more active screening for a genetic aetiology, the proportion of truly “idiopathic” osteoporosis cases will decline.

The following clinical features of idiopathic osteoporosis have been described [96–98] whereby males and females are equally affected, a family history of osteoporosis is common, the age at diagnosis is approximately 35 years, fractures are usually multiple occurring over 5–10 years and involve sites rich in cancellous bone, such as the vertebrae, and the hip is affected in approximately 10% of affected individuals.

## Evaluation of early-onset osteoporosis

### Medical history, physical examination and biochemical testing

Evaluation of low bone mass in children and young adults (Fig. 1) begins with obtaining medical history (e.g. personal and family history, fracture history, medications, chronic diseases, lifestyle factors) and performing physical examination (e.g. anthropometry, joint mobility, scoliosis, limb deformities, functional tests) and laboratory testing with the goal of searching for potential secondary causes [99]. A secondary cause of osteoporosis can be found in a substantial proportion of subjects [97]. Those with a fragility fracture require evaluation for secondary causes even in the absence of low BMD. Subjects who have suspicious findings on history and physical examination, and/or abnormalities on the basic laboratory testing, require additional laboratory tests (Table 4).

**Table 4:** Laboratory testing in serum or urine for searching of secondary causes in children and young adults

**Basic laboratory testing** Blood cell count Calcium, albumin, phosphate, ALP (total and bone specific) 25-OH Vitamin D, PTH Creatinine ESR TSH, fT4, fT3 24 h urine calcium and creatinine (in children spot urine)
**Additional laboratory testing** Bone turnover markers (e.g. PINP, CTX) Fasting glucose, HbA1c IGF1 Iron, ferritin, AST, ALT, tTG-IgA antibodies, anti-DGP- IgG antibodies LH, FSH, E2 LH, FSH, testosterone, SHBG Morning cortisol, ACTH, midnight cortisol, UFC, DST Prolactin Protein immunoelectrophoresis in serum/urine Tryptase

*ACTH* adrenocorticotropic hormone, *ALP* alkaline phosphatase, *ALT* alanine transaminase, *AST* aspartate transaminase, *CTX* C-terminal telopeptide, *DGP* deamidated gliadin peptide, *DST* dexamethasone suppression test, *E2* oestradiol, *ESR* erythrocyte sedimentation rate, *FSH* follicle-stimulating hormone, *fT3* free T3, *fT4* free T4, *HbA1c* glycosylated haemoglobin, *25-OH Vitamin D* 25-hydroxy vitamin D, *IGF1* insulin-like growth factor 1, *LH* luteinising hormone, *PINP* procollagen type I N-terminal propeptide, *PTH* parathyroid hormone, *SHBG* sex hormone-binding globulin, *TSH* thyroid-stimulating hormone, *tTG* tissue transglutaminase, *UFC* 24 h urinary free cortisol

Serum or urinary bone turnover markers (BTM) may provide useful information. If markers of resorption are elevated above the premenopausal range, excessive bone resorption is likely. However, the range of normal is wide, making interpretation difficult [100]. Bone resorption markers must be interpreted according to the patient’s age. Young adults are characterised by active bone remodelling and physiologic increases in BTMs [101, 102]. Additionally, elevated BTMs are observed after a recent fracture. Importantly, BTMs are more helpful in adults in monitoring disease course and treatment response.

### Genetic testing: the key to unresolved cases

Genetic studies have provided valuable information on bone biology, pathophysiological processes governing disease development and progression, and the genetic architecture of bone mass disorders. Monogenic disorders, such as early-onset osteoporosis, are more likely to arise from rare, highly penetrant genetic alterations inherited in an autosomal (dominant or recessive) or X-linked manner that ultimately result in aberrant protein function [63]. The classical approach to identify candidate gene variants in affected singletons or multiplex families with an apparent monogenic bone mass phenotype is by Sanger sequencing which is still considered the gold standard of clinical diagnostic testing. Single nucleotide substitutions (missense, nonsense and splicing) and small insertions or deletions (creating frameshift variations) in known genes (e.g. *LRP5, PLS3, WNT1, SGMS2*) are clearly identified in this yet time-consuming and costly hypothesis-driven method. HTS in the form of targeted gene panels, whole-exome sequencing (WES) and whole-genome sequencing (WGS) has been instrumental in gene and variant identification of monogenic osteoporosis, improving on throughput, turnaround time and costs [63]. Yet, it is important to keep in mind that the gene panels used in clinical practice are often limited and may not be up to date, considering the rapidly expanding spectrum of monogenic osteoporosis. The current Nosology of Genetic Skeletal Disorders includes tens of genes and conditions that may be relevant [64]. With increasing access to reasonably priced exome analyses and even WGS, there is probably going to be a shift from gene panels to other methods, particularly long-read sequencing which is better adapted at identifying structural variants [103]. Indeed, gene defects may involve deletions or duplications that can be easily missed when using diagnostic gene panels and short-read sequencing. Several cases of copy number variation (CNV)-related osteoporosis have been reportedly linked to, for example, type I collagen genes and *PLS3* [85, 104–106].

### Bone imaging

DXA is the preferred method for assessing bone mineral content (BMC) and areal BMD in children [23, 24]. The posterior–anterior spine and total body less head (TBLH) are the preferred sites for BMC and areal BMD measurements in most paediatric subjects. Other sites (e.g. proximal femur, lateral distal femur, distal radius) may be useful depending on each individualised case. A scan in children and young adults is usually indicated after two or more fragility fractures, after a fracture at an unusual site (such as the spine or hip), or in the presence of a chronic illness or medication predisposing to osteoporosis [27, 39]. If a follow-up DXA scan is indicated, the minimum interval between scans is 6–12 months. DXA uses very low radiation and is also fast and fully automated [99]. However, DXA is a 2D examination and it does not provide information on bone microarchitecture or differentiate between trabecular and cortical compartments. Additionally, DXA BMD can be falsely increased by collapsed vertebrae or mineral deposits at sites. Importantly, interpretation of DXA images in children requires adjustment not only for age and sex, but also for body or bone size, and skeletal maturity (bone age or pubertal status).

DXA vertebral fracture assessment (VFA) of the thoracic and lumbar spine may be used as a substitute for spine radiography in the identification of symptomatic and asymptomatic vertebral fractures in paediatric patients [23, 24]. Then, the Genant semi-quantitative method should be used. Important advantages of the VFA are the lower radiation exposure compared to plain radiographs, and the combination of BMD and VFA information through performing the same examination. Quantitative computed tomography (QCT), pQCT (peripheral QCT) and HR-pQCT (high resolution QCT) are research techniques used to characterise bone deficits in children. They can be used clinically in these populations where appropriate reference data and expertise are available.

### The power of bone biopsies

Bone biopsies could hold the key to diagnosing unclear and potentially complicated cases of young individuals presenting with early-onset osteoporosis. Information on the rate of bone resorption and remodelling, degree of mineralisation (hypo- vs hypermineralisation) defects, bone structure and material properties, and chronic comorbidities (e.g. presence of multiple myeloma) can be unveiled that consequently will aid in differential diagnosis and patient management, especially treatment. Labelling of the bone with a double or quadruple tetracycline that binds to the mineralised bone surface is recommended to calculate the rate of bone formation and turnover [107], and in so doing characterise different low bone mass causes (e.g. low-turnover osteoporosis versus osteomalacia). Anterior iliac crest is the preferred sampling site thanks to its accessibility, which circumvents the need for surgery [108]. Yet, routine use of this invasive procedure remains low in the clinical setting [34].

Other tools can be used to further analyse the sampled bone tissue providing data on the mineralised bone volume and extracellular matrix, bone properties and mechanical strength and osteocytes lacunae. Such tools include quantitative backscatter electron imaging (qEBI), small-angle X-ray scattering, vibrational spectroscopy, nanoindentation and X-ray tomography, reviewed in detail elsewhere [34]. Histomorphometry using Masson–Goldner trichrome staining enables tissue and morphological identification and helps quantify osteoid and mineralised bone. The presence of defective collagen fibrils, altered cross-linking or thinner fibrils can also be observed in the same stained tissue sections and may help distinguish different pathologies, including OI types [109–111].

## Treatment options for early-onset osteoporosis

**Fig. 2:**
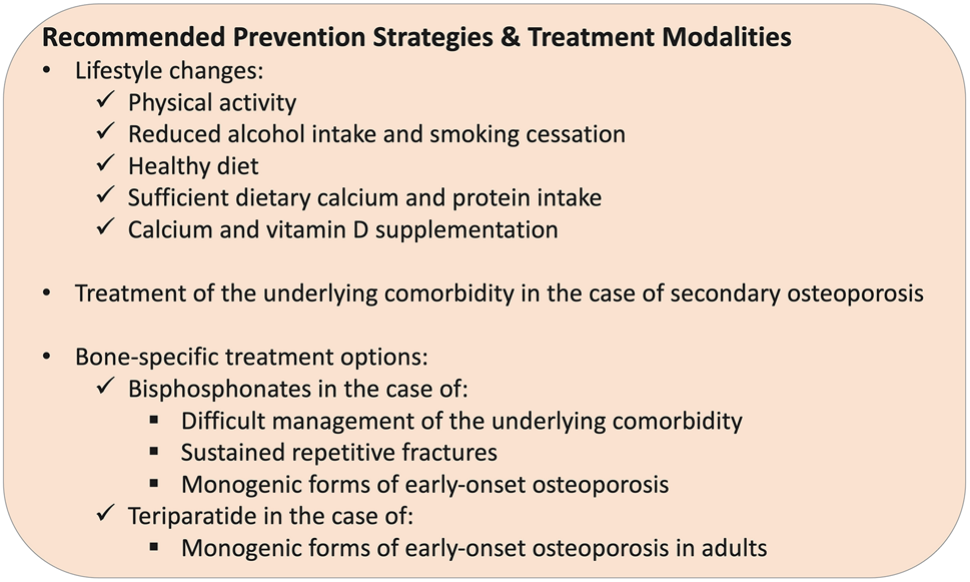
Proposed prevention strategies and targeted treatment options for children and young adults with early-onset osteoporosis

The low prevalence of children and young adults with early-onset osteoporosis has made it difficult to undertake large-scale clinical trials, particularly to investigate the effect of pharmacological intervention on fracture prevention. For this reason, there are currently no evidence-based guidelines for the treatment of affected individuals with early-onset osteoporosis. Instead, preventive measures and individualised treatment approaches are generally recommended (Fig. 2), as discussed below.

Proper supplementation of calcium and vitamin D should be given, especially in case of deficiency or insufficiency following laboratory investigations [17, 112, 113]. Lifestyle changes are advised in the form of increased physical activity, reduced alcohol intake, no smoking and sufficient protein intake, which have indeed resulted in improved BMD status in young affected individuals [114, 115]. Treatment of the underlying comorbidity is a must which will also have beneficial effects on bone health, for example, gluten-free diet in coeliac disease, treatment of amenorrhoea with oestrogen replacement therapy, treatment of inflammatory bowel disease and rheumatoid arthritis with anti-TNF alpha antibodies, etc. [39]. When treatment of the chronic disease is not feasible or repetitive fractures are sustained, antiresorptive or osteoanabolic therapy is considered. Increase in BMD following bisphosphonate treatment has been reported in young individuals with secondary osteoporosis [27], including patients with anorexia nervosa treated with risedronate [116], women with ovarian failure after allogeneic stem cell transplant treated with risedronate and zoledronic acid [117], individuals with cystic fibrosis treated with alendronate [118] or zoledronate [119], as well as Crohn’s disease [120] and ß-thalassaemia major [121], amongst others. However, bone pharmaceuticals in other risk groups might not be favourable (e.g. pregnancy and women of childbearing age) due to potential adverse effects. In summary, although most studies have demonstrated an improvement in BMD, studies that focus on decreased fractures as the primary outcome are yet to be conducted.

Treatments of monogenic forms of early-onset osteoporosis have also been investigated, but not extensively. Teriparatide treatment showed increased bone turnover in individuals with missense variants in *LRP5*, *LRP6* and *WNT1* [122, 123], and splicing variants in *PLS3* [123]. Improvement in BMD Z-scores with reshaping of compressed vertebrae was also observed in zoledronate-treated individuals harbouring a large fragment deletion variant in *PLS3* [106]. Positive outcomes were seen in patients with deleterious *SGMS2* variants following bisphosphonate therapy, including improvement in back pain and quality of life, and fracture prevention [87]. However, the same cannot be said for individuals with *WNT1* and *LRP5* variants who exhibited no effect after bisphosphonate treatment [77, 124]. In conclusion, more large-scale and long-term studies are required to properly evaluate the effects of different antiresorptive and osteoanabolic treatment, including anti-sclerostin therapy and possible combinatory treatment modalities not just on BMD, but even fracture risk.

### Concluding remarks

Early-onset osteoporosis, although rare, remains a significant disorder with considerable morbidity that presents with diagnostic challenges. If no genetic causal variants are identified following high-throughput DNA sequencing, then transcriptomics, metabolomics and proteomics should be considered enabling a multi-omics approach that can be coupled with machine learning tools. Identification of the underlying cause can inform about inheritance patterns, treatment options and patient monitoring, all of which are also beneficial to other potentially susceptible relatives. The need for collaborations between clinical, basic and translational researchers through international scientific consortia (e.g. GEFOS: http://www.gefos.org and GENOMOS: http://www.genomos.eu), COST Actions (e.g. GEMSTONE COST Action, CA18139: https://cost-gemstone.eu), European Reference Networks (e.g. European Network for Rare Bone Conditions, ERN BOND: https://ernbond.eu), rare bone disorder registries (e.g. Osteogenesis Imperfecta: https://oif.org/oiregistry), as well as patient organisations has become more evident to overcome diagnostic obstacles and provide timely care to patients.

The canonical WNT signalling pathway is presently regarded as a key regulator of bone metabolism. Its role in bone was discovered by studying monogenic diseases with low and high bone mass. These genetic and molecular discoveries led to the development of a new anabolic osteoporosis medication, sclerostin antibody [125]. Similarly, genetic and molecular discoveries in other rare genetic bone mass disorders such as pycnodysostosis (cathepsin K antibody), juvenile Paget’s disease (RANKL antibody), hypophosphataemic rickets (burosumab) and hypophosphatasia (asfotase alfa) have been of key importance in drug development [126–129].

It is likely that significant scientific advancements can still be made by studying patients and families with early-onset osteoporosis, leading to renewal of our understanding of bone metabolism and pathogenesis of skeletal fragility. In the long-term, research discoveries are likely to enable the development of new modes of osteoporosis therapy and provide new tools for improved diagnostics and follow-up of affected individuals.

## Data Availability

Not applicable.
